# In-Situ Forming Polyester Implants for Sustained Intravesical Oxybutynin Release

**DOI:** 10.3390/pharmaceutics17111369

**Published:** 2025-10-23

**Authors:** Michael Uwe Hartig, Jan Appelhaus, Marc Vollenbröker, Alf Lamprecht

**Affiliations:** 1 Department of Pharmaceutics, Institute of Pharmacy, University of Bonn, 53121 Bonn, Germany; s6mlhart@uni-bonn.de (M.U.H.); j.appelhaus@uni-bonn.de (J.A.); 2 Farco Pharma GmbH, 50670 Cologne, Germany

**Keywords:** intravesical delivery, oxybutynin, PLGA, biodegradable, in vitro, in situ forming implant

## Abstract

**Background/Objectives:** Neurogenic detrusor overactivity (NDO), caused by spinal cord injury or multiple sclerosis, is marked by involuntary bladder contractions and reduced urine volume. Current therapy requires frequent catheterization with oxybutynin hydrochloride. This work investigates a novel in situ forming implant (ISFI) with PLGA as a sustained-release formulation for the urinary bladder by quantifying drug release, polymer degradation, and solvent release in vitro. **Methods/Results:** Various formulation parameters were investigated, of which the drug load and PLGA termination were found to have the highest impact on drug release and polymer degradation. An increase in drug load from 1.5% to 7.5% for implants with the ester-terminated PLGA enhanced the degradation from 0% to around 20% after 7 d. Oxybutynin base catalyzed the polymer degradation, as implants with PLGA 502 and 15% drug load exhibited a degradation of 33% compared to 0% for 1.5% drug load. In the case of 1.5% drug load, the degradation could be increased by the use of an acid-terminated PLGA, compared to an ester-terminated. **Conclusion:** In summary, the feasibility of a biodegradable ISFI for NDO patients was shown, which could allow a single administration up to approx. one week, improving the quality of life for NDO patients. Additionally, this work provided insight to which formulation parameters can help to parallel drug release and polymer degradation.

## 1. Introduction

In situ forming implants (ISFIs) are a nascent strategy for sustained drug delivery and present a promising alternative to conventional systems, such as microparticles or solid intravesical implants [1]. ISFIs are administered as a liquid and undergo a sol-to-gel or sol-to-solid phase transition in situ, typically triggered by solvent exchange with aqueous body fluids. For intravesical applications, ISFIs must fulfill additional requirements. Specifically, they must remain in the bladder post-voiding, release the drug over a defined period, and degrade completely to avoid accumulation within the organ.

The urinary bladder poses distinctive challenges as a potential drug delivery site. Frequent micturition rapidly flushes out liquid formulations, necessitating repeated catheterization, often up to five times daily [2]. Furthermore, the bladder environment is dynamic, with fluctuations in pH, urine volume, and hydrodynamics. These fluctuations affect the release behavior and biodegradation kinetics of implant materials. These specific conditions can induce degradation pathways that are distinct from those observed in muscle or subcutaneous tissues, highlighting the need for tailored biodegradable systems.

Poly(lactic-co-glycolic acid) (PLGA) is a prominent example of a biodegradable polymer, distinguished by its tunable degradation characteristics, biocompatibility, and regulatory approval [3,4]. The degradation of the polymer occurs via hydrolysis, resulting in the production of lactic and glycolic acid. The rate of degradation is influenced by various factors, including the polymer composition, specifically the ratio of lactic to glycolic acid, as well as the end-groups, such as the presence of free acid or end-capped groups. Additionally, the molecular weight of the polymer plays a role in its degradation rate. Furthermore, environmental factors such as pH (e.g., alkaline-catalyzed hydrolysis) and autocatalysis due to the accumulation of degradation products can significantly accelerate breakdown [5,6]. Two widely used PLGA grades are Resomer^®^ RG 502 and RG 502H. These polymers possess a molecular weight ranging from 7000 to 17,000 g/mol and exhibit a 50:50 ratio of lactic acid to glycolic acid. RG 502H is characterized by an acid termination, in contrast to RG 502, which is distinguished by an ester termination. The presence of the hydrophilic carboxylic end group has been demonstrated to expedite the hydrolysis process in RG 502H [7]. A review of the existing literature reveals that both polymers have been the subject of investigation and utilization in the context of in situ forming implants with ketoprofen [8].

The release of the drug from PLGA-based ISFIs can be characterized by an initial burst release, followed by diffusion-controlled release and eventual polymer erosion [9,10]. These characteristics are only partially compatible with the use of PLGA-based ISFIs for sustained intravesical delivery, with the potential to extend dosing intervals to once weekly, thereby improving patient compliance and minimizing catheterization frequency. It is evident that the primary objective is to minimize the burst release.

This strategy is particularly relevant for treating neurogenic detrusor overactivity (NDO), a condition resulting from multiple sclerosis or spinal cord injury that leads to involuntary bladder contractions and loss of voluntary control [9,10,11,12]. Oxybutynin hydrochloride, a widely used anticholinergic, is effective when administered orally (e.g., Ditropan^®^). However, systemic side effects, such as constipation, dry mouth, and dizziness, often lead to treatment discontinuation. Intravesical delivery provides a localized alternative with fewer side effects [13]. However, at present, there is only one formulation on the market that provides local drug availability via bladder administration as a solution [14].

Furthermore, there is an absence of a biodegradable sustained-release intravesical dosage form that is capable of maintaining therapeutic levels over an extended period. Non-degradable systems, such as the TAR-200 silicone-based device under investigation for non-muscle-invasive bladder cancer, require cystoscopic removal [15]. The utilization of ISFIs could offer certain advantages, including their capacity for biodegradability and their minimally invasive administration.

In this study, the potential for intravesical administration of PLGA-based ISFIs containing oxybutynin is explored. The formulation is designed to administer the drug over a period of seven days, a clinically relevant interval that balances treatment efficacy with patient convenience. The present study systematically evaluated the influence of PLGA type (502 vs. 502H), polymer concentration, drug loading, and medium pH on in vitro drug release and polymer degradation.

## 2. Materials and Methods

### 2.1. Materials

Oxybutynin hydrochloride was kindly provided by Klosterfrau Healthcare Group (Berlin, Germany) and was converted into its free base form. Poly (L-lactic-co-glycolide acid) (PLGA 50:50) Resomer^®^ RG 502 and Resomer^®^ RG 502H were purchased from Evonik AG (Ingelheim, Germany). Tetrahydrofurfuryl alcohol polyethylene glycol ether (GF), was purchased from Sigma Aldrich (Steinheim, Germany). All other chemicals used for formulation design and HPLC analysis were of analytical grade or higher.

### 2.2. Preparation of Oxybutynin Base

15 g oxybutynin hydrochloride was dissolved under stirring in 200 mL MilliQ water. After the addition of 1 g sodium hydroxide to adjust the pH to 10, the free base of the drug precipitated. The drug was extracted using a separating funnel and chloroform as the solvent for oxybutynin base. The chloroform was removed using a rotary evaporator at 48 °C and 600–650 mbar. In a vacuum dryer at 40 °C for 24 h, the remaining solvent was removed. A yield of 90% was achieved.

### 2.3. Preparation of ISFI-Solutions

For the preparation of the ISFI solutions, oxybutynin hydrochloride or oxybutynin base (1.5%, 3.0%, 7.5%, and 15% *w*/*w*) was dissolved under continuous stirring in GF for 24 h. Resomer^®^ RG 502 or Resomer^®^ RG 502H (30% and 15% *w*/*w*) was added to the drug solution and stirred for further 24 h. Finally, the solution was subjected to ultrasonication in an ultrasound water bath sonorex digitec DT 106 from Bandelin (Berlin, Germany) for 2 h at 120 W and a frequency of 35 kHz.

### 2.4. Viscosity of ISFI-Solutions

Viscosity measurements were performed using the rotational viscometer MCR92 from Anton Paar (Graz, Austria). ISFI solutions were analyzed utilizing a cone-plate geometry (CP-50-1). The plate had a diameter of 49.965 mm and the cone angle was 1.001°. Solutions were analyzed at a plate temperature of 25 °C. With a cone truncation of 0.097 mm the shear rate was increased from 1 s^−1^ to 200 s^−1^, where the final viscosity was measured.

### 2.5. Differential Scanning Calorimetry

Differential Scanning Calorimetry (DSC) measurements were carried out on a Mettler-Toledo DSC 2, which was equipped with a nitrogen cooling system from Mettler-Toledo (Giessen, Germany). Glass transition temperatures (T_g_) were determined by using a heating rate of 2 K/min starting at 0 °C until 80 °C were reached. For the evaluation of the glass transition temperature a multi-frequency temperature modulation (TOPEM mode) was used. In total, 10 mg of sample was weighed into aluminum pans and sealed with a lid.

### 2.6. In Vitro Drug-Release Testing

Drug release from the formulations was assessed using a shaking bath type 1083 from GFL Technology (Burgwedel, Germany) with a shaking frequency of 64 min^−1^. Temperature was set to 37 °C to mimic the temperature inside a urinary bladder. ISFI solutions were injected into glass vials containing 20 mL artificial urine as release medium. A sample of 1 L of artificial urine consisted of 24.25 g urea and 14.84 g tris sodium phosphate dodecahydrate and was adjusted to pH 4.0, 6.0 and 8.0 with hydrochloric acid. Samples of 0.4 mL were taken after 1 h, 2 h, 4 h, 8 h, 12 h, 24 h and the removed volume was replaced with fresh release medium. After 24 h another sample was taken before the complete medium was replaced with artificial urine in order to simulate urination. This pattern of sampling and simulated urination was continued for a total of 7 days. Samples taken were analyzed by high-performance liquid chromatography (HPLC).

### 2.7. Degradation of PLGA

The release medium collected daily was analyzed for the monomer lactic acid. As preparation for the HPLC, the pH of the release medium was adjusted with 1 mL concentrated hydrochloric acid to ensure that the lactic acid is present as a monomer. To increase the degradation of lactic acid oligomers or PLGA microparticles, that were created by injection of the ISFI solution to the release medium, the samples were stored in a drying cabinet for 5 days at a temperature of 60 °C. After that procedure, the samples were analyzed for lactic acid content by HPLC.

### 2.8. High-Performance Liquid Chromatography Analysis

HPLC analysis for oxybutynin base and lactic acid were performed on a Shimadzu, LC-2030C 3D Plus (Kyoto, Japan). For detection, the photodiode array detector was set to 203 nm. The column temperature was set to 40 °C. A RP18 column (LiChrospher^®^ 100 RP 18–5 µ EC, 250 × 4.6 mm) with a RP18 pre-column (LiChrospher^®^ 100 RP 18–5µ EC, 10 × 4 mm) from Merck KGaA (Darmstadt, Germany) was used for the analysis of oxybutynin base. The mobile phase consisted of MilliQ with 0.2% (*v*/*v*) TEA adjusted to a pH of 3.0 with ortho-phosphoric acid and acetonitrile (40:60% *v*/*v*) [16]. In addition, the isocratic flow was set to 1.0 mL/min and the injection volume was 10 µL. A LOD of 0.35 µg/mL and a LOQ of 1.07 µg/mL were determined. The specificity was ensured by injection of oxybutynin standard from artificial urine with different pHs. No peaks interfered with the oxybutynin peak. Following parameters were evaluated: slope = 14465; y-intercept = 150.76; R^2^ = 0.9994; linear range = 1–100 µg/mL; retention time: 6.0 min.

In case of the analysis of the monomers of lactic acid a RP18 column (EC NUCLEODUR C18 Pyramid 5 µm, 250 × 4.6 mm) with a RP18 pre-column (EC 4/3 NUCLEOSIL 100-5 C18) from Macherey-Nagel GmbH & Co. KG (Düren, Germany) was used. As mobile phase MilliQ water was used with the addition of 0.2% (*v*/*v*) TEA and ortho-phosphoric acid to achieve a pH of 3.0. The flow rate was set to 0.7 mL/min and the injection volume was 30 µL. A LOD of 4.37 µg/mL and a LOQ of 13.25 µg/mL were determined. Its specificity was ensured by the injection of a lactic acid standard from artificial urine with different pH and artificial urine incubated with HCl for 5 days at 60 °C. No peaks interfered with the lactic acid peak. The following parameters were evaluated: slope = 1944.32; y-intercept = −1277.05; R^2^ = 0.9970; linear range = 13–200 µg/mL; retention time = 6.9 min.

### 2.9. Gas Chromatography (GC)

GC analysis of GF release from the implant was performed using an Agilent 6890N gas chromatograph from Agilent Technologies Inc. (Santa Clara, CA, USA) equipped with an Agilent 7683 auto sampler and a FS-CS-624 30 m × 0.25 mm ID × 1.8 µm capillary column from CS—Chromatographie Service GmbH (Langerwehe, Germany). Nitrogen was used as the carrier gas at a flow rate of 2 mL/min. The injector was set to 250 °C and a volume of 0.2 µL was injected each run. The injector operated in splitless mode with a purge time of 1.3 min. The initial oven temperature of 80 °C was maintained for 1.5 min before increasing by 10 °C/min to 150 °C. This temperature was maintained for 5 min before rising by 5 °C/min to 200 °C, which was maintained for 20 min. Finally, the temperature was increased by 20 °C/min to 235 °C for an additional 8 min and a total run time of 53.25 min. Samples were quantified by a flame ionization detector set to 270 °C.

### 2.10. Visualization of Implants

For a better visualization of the occurrence of in situ forming implants during the release study, pictures of the ISFI were conducted directly after the injection of the ISFI solution into the artificial urine. After 7 d pictures of the same implants were made for a comparison between the initial and final occurrence.

### 2.11. Statistical Evaluation

Experiments were conducted at least as triplicate. GraphPad Prism 8 software was used for statistical analysis. Mann–Whitney U tests and Kruskal–Wallis–Dunn’s tests were performed with significance levels of: * *p* < 0.05; ** *p* < 0.01; *** *p* < 0.001; **** *p* < 0.0001. Statistical analysis for the drug release was performed for the timepoints 1 d and 7 d. In addition, the lactic acid content after 7 d and the solvent release after 1 d and 7 d was statistically analyzed. Drug release and solvent release were differentiated into “initial” phase, which was defined as 0 h–24 h and “late” which was defined as the time between 24 h and 168 h.

With the help of MODDE 13.1 software from Sartorius AG (Goettingen, Germany) possible correlations between different formulation aspects and the chosen responses were calculated. The PLGA termination (acid or ester), polymer load and drug load were chosen as factors, while viscosity, initial and final drug release, initial and final solvent release and degradation of the polymer were chosen as responses.

## 3. Results

### 3.1. Viscosity of ISFI Solutions

**Figure 1:**
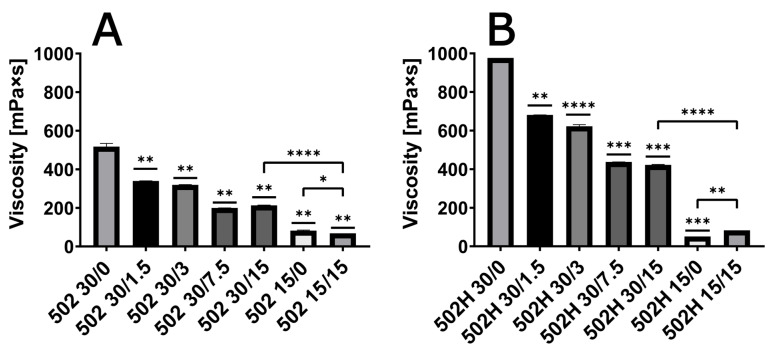
Viscosity of ISFI solutions with a polymer load of 15% or 30% and a varying oxybutynin base content (0%, 1.5%, 3%, 7.5% and 15%) with RG 502 (**A**) and RG 502H (**B**). ISFI solutions were compared with the 30/0 as a reference if not indicated differently (* *p* < 0.05; ** *p* < 0.01; *** *p* < 0.001; **** *p* < 0.0001; *n* = 3).

A comparison of ISFI solutions prepared from PLGA grades 502 and 502H reveals a significant difference in viscosity (see Figure 1). The utilization of 502H in comparison to 502 resulted in an augmentation of viscosity by a factor of two. A consistent decline in viscosity was observed as the amount of active ingredient increased, up to a maximum drug load of 7.5%, irrespective of the polymer grade. However, this effect was not observed when working with lower polymer concentrations, as evidenced by the 15% PLGA content.

### 3.2. Glass Transition Temperature of ISFI

In addition to the viscosity, the T_g_ provided further insight into the impact of increased drug loading on implant structure (see Table 1). Consequently, a decrease in the T_g_ was observed after seven days of release testing with increasing drug content. A substantial alteration in T_g_ was not detected by the PLGA termination or polymer content.

### 3.3. Impact of Variable pHs of Artificial Urine

**Figure 2:**
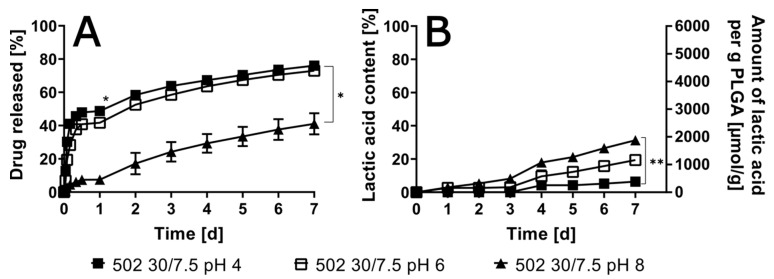
In vitro drug release for 502-30/7.5 in artificial urine at pHs of 4, 6 and 8 with oxybutynin base (**A**) as well as lactic acid in the release medium and amount of released lactic acid per g polymer implant for the same formulation are given over 7 days (**B**); (*n* = 3; * *p* < 0.05; ** *p* < 0.01).

Preliminary analysis revealed that oxybutynin, when formulated as a free base, exhibited a markedly diminished burst release in comparison to its hydrochloride salt formulation (see Figure S1). It is imperative to examine the pH-dependent release behavior, as it is instrumental in determining the pH of the release medium and its impact on the drug release kinetics. This examination was conducted within the physiologically relevant range of pH 4 to 8 for an ISFI with RG 502 (30% polymer content and 7.5% drug load, Figure 2). The burst release, which occurred within 24 h, was distinctly visible at pHs 4 and 6, while being nearly absent at pH 8. However, the continuous release until day 7 was comparable in all three cases. Conversely, polymer degradation was accelerated with increasing pHs, although this phenomenon occurred at a rate 2 to 3 times slower than the drug release.

### 3.4. Variation of Drug Load in the ISFIs

**Figure 3:**
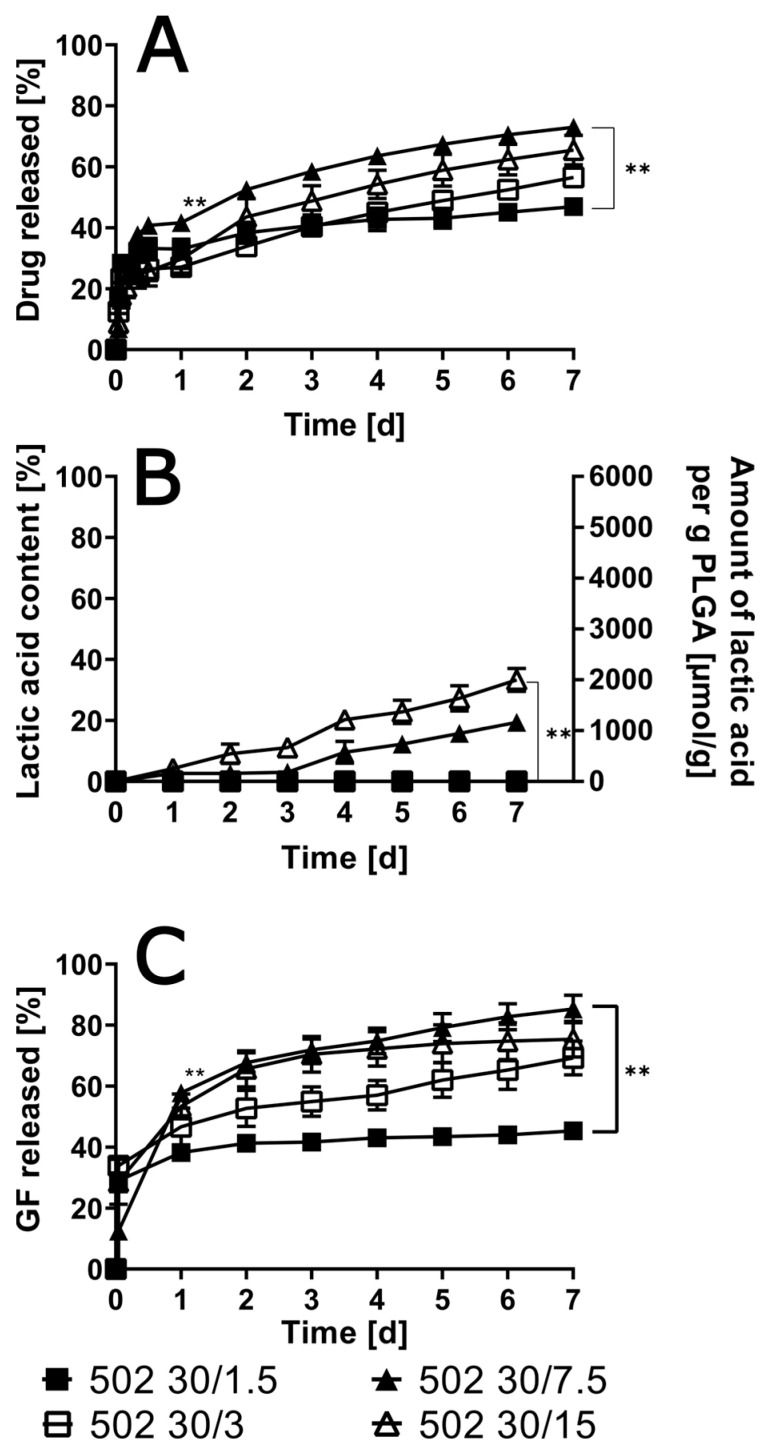
In vitro drug release for different oxybutynin base loads in ISFI formulations in artificial urine (pH 6) (**A**) as well as lactic acid (**B**) and solvent (**C**) in the release medium are given over 7 days (*n* = 3; ** *p* < 0.01).

A correlation was observed between the final cumulative amount of drug released after 7 days and the initial drug load. As the dosage of the pharmaceutical compound increased, the final cumulative amount of the drug released after seven days increased until it reached a dosage of 7.5%. Subsequent to the administration of a drug load that constituted 7.5% of the total dosage, there was no further augmentation in the release (see Figure 3). However, a general tendency of lower drug loads leading to slower release was observed. This finding indicates that the release from ISFIs containing 1.5% of the drug is 1.5-fold slower than the release from ISFIs containing 7.5% of the drug.

While the solvent release exhibited a significantly accelerated rate in comparison to the drug release, the occurrence of accelerated solvent release was also found to be drug-load dependent. This accelerated solvent release was observed to be more pronounced in cases involving higher drug loads. It is noteworthy that the amount of solvent released was nearly twofold lower for 1.5% compared to 7.5% drug load.

Polymer degradation was also accelerated by higher drug loads, where the highest lactic acid amount was 33.3% ± 3.7% for the implants with a drug load of 15%, showing a significant difference to the ISFI with 1.5% and 3% drug load after 7 days. In this instance, linear curve progressions were observed.

### 3.5. Influence of Polymer Grade and Content

**Figure 4:**
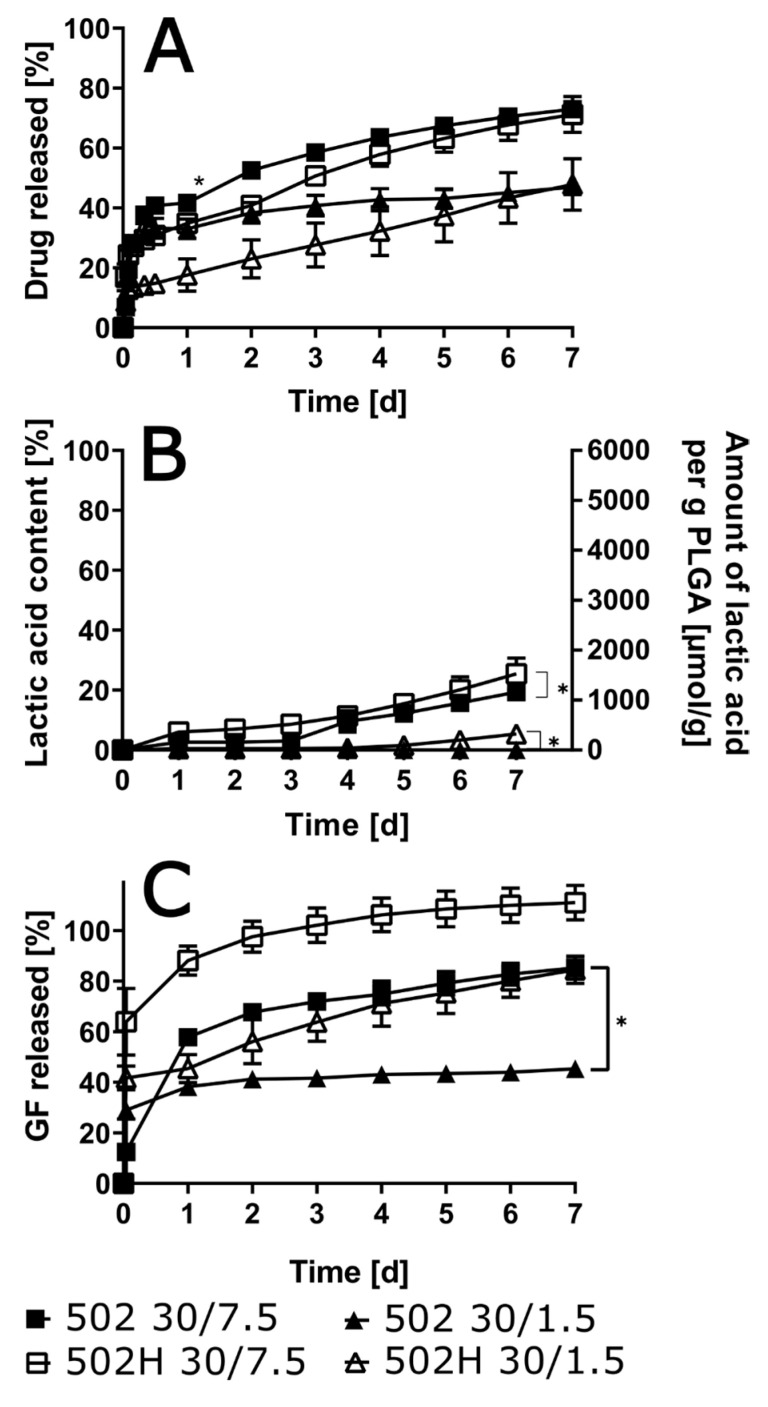
In vitro drug release for different ISFI formulations with varied polymer grade and oxybutynin base load in artificial urine (pH 6) (**A**) as well as lactic acid (**B**) and solvent (**C**) in the release medium are given over 7 days (*n* = 3; * *p* < 0.05).

A substantial decrease in burst release was observed in the 502 group, in contrast to the findings in the other combinations (see Figure 4). This outcome is indicative of a significant reduction in drug content. Following a period of seven days, the final levels of the released drug exhibited a greater influence from the drug content than from the polymer type. This resulted in approximately 65% of the drug being released for the 7.5% drug load, and slightly higher values, approximately 40%, for the 1.5% drug load.

The degradation rate of the polymer was marginally augmented in the presence of 502H in comparison with 502. Nevertheless, the impact of the drug load proved to be more pronounced than that of the polymer type. Initial findings indicated that the 502H variant exhibited faster solvent release; however, subsequent analysis revealed that the drug load exerted a more substantial influence on these results compared to the polymer type. It is noteworthy that with low drug load and end-capped PLGA, more than 50% of the solvent remained within the ISFI over the entire experimental period of 7 days.

**Figure 5:**
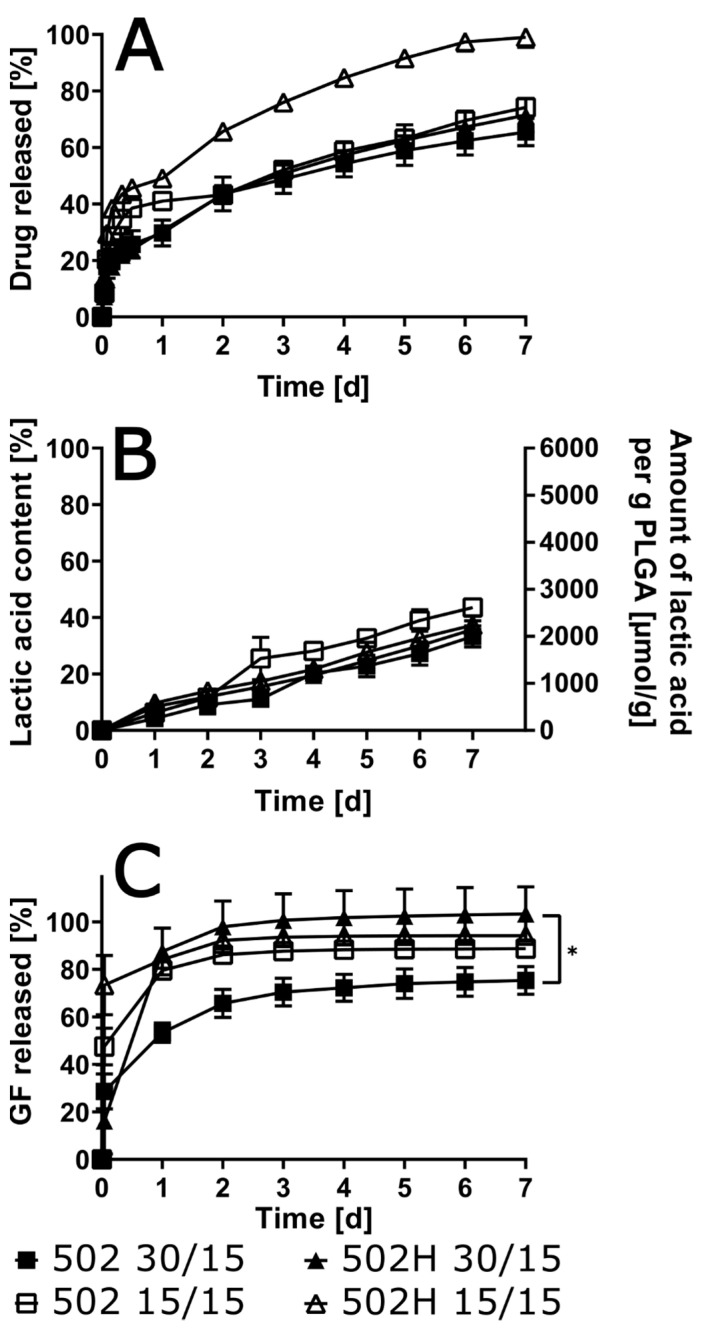
In vitro drug release for different ISFI formulations with oxybutynin base, varied polymer grade and polymer load (15% vs. 30%) in artificial urine (pH 6) (**A**) as well as lactic acid (**B**) and solvent (**C**) in the release medium are given over 7 days (*n* = 3; * *p* < 0.05).

A decline in polymer content within the ISFI typically resulted in an enhancement of burst release, irrespective of the utilized polymer (Figure 5). However, the impact of polymer concentration on the final cumulative drug release was only observed with 502H: a reduction in polymer load resulted in a higher final cumulative oxybutynin release of 99.0% ± 3.6% compared to implants with 30% polymer load (71.5% ± 5.9%). Contrary to expectations, release experiments with 502 demonstrated negligible differences when the polymer content was reduced from 30% to 15%. With regard to polymer degradation, neither the polymer content nor the polymer type exhibited a significant impact. The solvent release was found to be distinctly impacted by the polymer concentration. While low polymer content (15%) resulted in comparable solvent release for both polymer types, high polymer content (30%) resulted in distinctly quicker and complete solvent release with 502H.

All implants were visualized after injection into artificial urine pH 6 and after 7 d in the release medium (see Figures S2–S21). A modification in the drug load resulted in alterations to the implant’s macroscopic morphology. Specifically, implants with a 1.5% drug load exhibited a more solid structure immediately following injection. Conversely, an elevated drug load resulted in a more pliable structure, manifesting as a floating semisolid consistency following injection, particularly at a 15% drug load. A discrepancy in morphology was identified between implants with acid-terminated and end-capped PLGA after seven days, as implants with 502 exhibited a more refined surface texture.

**Figure 6:**
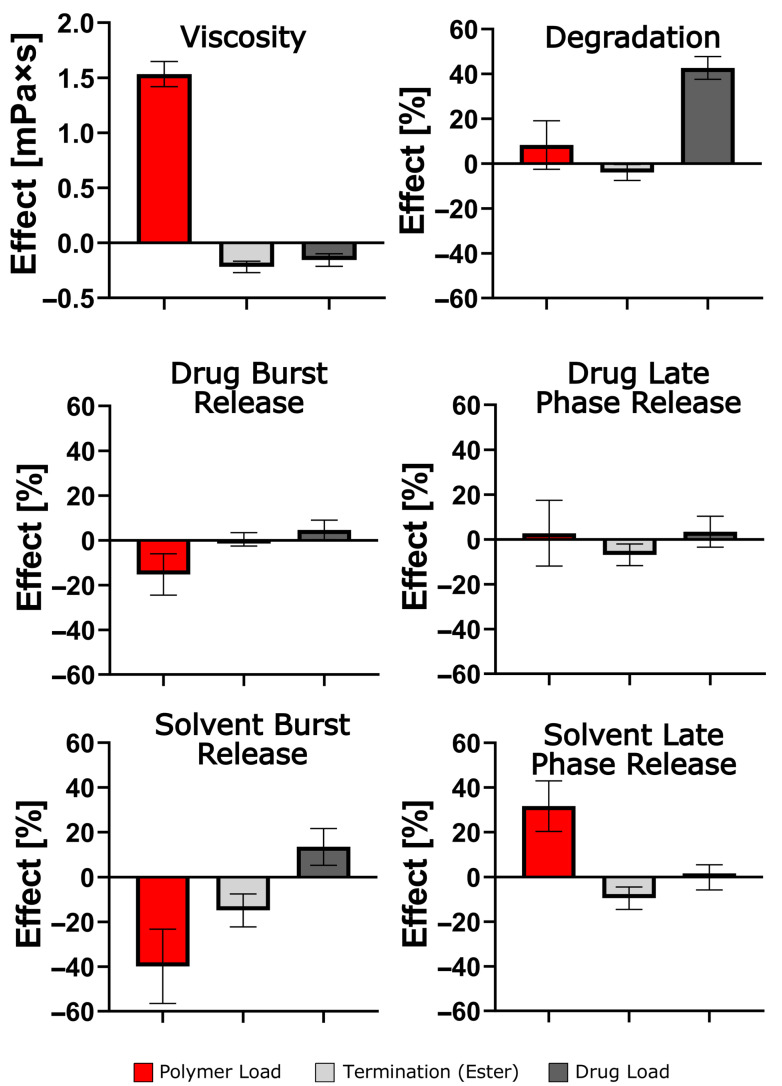
Evaluation based on MODDE^®^ of the impact of formulation parameters on viscosity of polymer solution, implant degradation, drug burst release (0 h–24 h), drug late phase release (24 h–168 h), or solvent burst release (0 h–24 h) and solvent late phase release (24 h–168 h). A model term is significant, when the standard deviation does not exceed the x-axis.

A statistical evaluation of the results identified polymer content as the most significant parameter, primarily due to its effect on the viscosity of the ISFI solutions (Figure 6). The subdivision of burst and late phase release (0 h–24 h or 24 h–168 h) was found to be an effective method of eliminating the otherwise dominant effect of a large burst release on total release after 7 days, for both drug and solvent, respectively. For instance, a decrease in polymer load resulted in a higher initial release; however, it did not affect the late phase release. This is in contrast to the termination of PLGA, which had no effect on the burst drug release but reduced the late phase drug release. The impact of the polymer grade was surprisingly limited, primarily manifesting as an effect on burst solvent release. It has been confirmed that the calculations conducted have substantiated a primary factor that significantly impacts the rate of polymer degradation in the presence of a given drug load.

## 4. Discussion

In contrast to non-biodegradable implants, such as TAR-200, which utilize a silicone matrix and necessitate invasive insertion and extraction, ISFIs with PLGA can naturally degrade, thereby eliminating the requirement for surgical intervention. This characteristic could contribute to improved patient outcomes and a higher level of acceptance within clinical practice [15]. During the preliminary formulation studies, PLGA grades 503 and 504 were evaluated for their suitability. However, despite the relatively extended release (>3 weeks), high drug loads, and the subsequent escalating risks associated with burst drug releases, the present study concentrated exclusively on PLGA grade 502 and 502H.

The formation and drug release behavior of ISFIs are critically dependent on solution viscosity, polymer content, and the effects of additives. A multitude of studies have indicated that accelerated solidification upon injection can be achieved with higher viscosity solutions. The underlying mechanism for this phenomenon involves the facilitation of rapid phase inversion and matrix formation [17,18,19]. Viscosity is primarily influenced by polymer load, polymer characteristics, and drug–polymer interactions [20,21,22,23]. A study was conducted to investigate the impact of the end group of poly(lactic acid) (PLA) on the formation of hydrogen bonds. The study concluded that the addition of an acid termination group to the polymer chain has the potential to enhance the strength of these bonds, thereby increasing the viscosity of the material. This finding is in accordance with the results reported in our own study [24]. As demonstrated in previous studies, a decrease in polymer load results in reduced viscosity and delayed solidification, thereby enhancing initial drug release [18,20,21]. As previously demonstrated in extant studies, a diminished polymer concentration or reduced molecular weight of the selected polymer results in a reduced degree of chain entanglement, thereby diminishing the viscosity. This phenomenon has been substantiated in our study, as evidenced by the polymer concentration [25,26]. This change in viscosity does not always lead to a change in degradation rate, only influencing the drug release [24]. As long as an intact polymer matrix is established, degradation is influenced more by other factors, such as the termination of PLGA [24]. This observation was replicated in the present study, wherein a decrease in polymer content was found to result in a reduction of viscosity, thereby impacting the initial drug and solvent release. However, this reduction in viscosity had no effect on the degradation rate. The extent of burst release is contingent upon factors such as viscosity, as well as the water miscibility of the solvent utilized. The employment of a solvent characterized by elevated water-miscibility levels has been demonstrated to enhance solvent exchange, thereby precipitating a heightened burst release in instances where the drug exhibits a high degree of solubility in the utilized solvent. In this instance, the oxybutynin base is soluble in glycofurol, and the solvent is characterized by a high degree of water miscibility. Therefore, a reduction in polymer content results in a slower phase inversion. The use of a water-miscible solvent with a drug that is soluble in it increases burst release [7]. As demonstrated previously, alterations in the PLGA end group particularly influence degradation and viscosity. The transition from acid- to ester-terminated PLGA has been shown to reduce viscosity, accelerate solvent exchange, and delay matrix solidification. This, in turn, results in more porous implant structure and facilitates early release [27]. An increase in water uptake, resulting from a more porous matrix, has been shown to enhance the hydrolytic degradation of acid-terminated PLGA. This enhancement is attributed to the increased hydrophilicity of the matrix. In this study, the impact of polymer type was less pronounced—a phenomenon that may be attributed to the substantial presence of acceptor phase and its pH level, which closely resembled urine conditions. This observation contrasts with findings from other studies.

The drug loading exhibited a significant impact on all investigated factors. The process of drug loading has been demonstrated to modulate both the physical properties and the chemical degradation of the resultant implant. The augmentation of drug loading has been demonstrated to reduce viscosity, thereby decelerating matrix precipitation and expediting the initial release of the drug and solvent. Furthermore, the plasticizing effect of oxybutynin on the viscosity of the ISFI solutions could be demonstrated by a reduction in T_g_ with increasing drug loading. Oxybutynin has been demonstrated to intercalate between polymer chains, thereby increasing chain mobility and reducing the glass transition temperature. This, in turn, has the potential to influence the viscosity of ISFI solutions [28]. The physicochemical properties of the API, particularly its alkalinity, profoundly influence long-term degradation. For instance, an alkaline microenvironment has been shown to promote the hydrolysis of PLGA and increase release rates [29,30,31]. However, it is important to note that excessive alkalinity may impose limitations on release due to solubility constraints, as evidenced by observations with oxybutynin base at elevated pH [32]. The burst release of oxybutynin is dependent on the surrounding pH of the release medium. At an acidic pH, a greater proportion of oxybutynin is protonated, thereby increasing its water solubility and enhancing its propensity to diffuse out of the polymer matrix. The use of Oxybutynin-HCl results in a higher protonated fraction of the drug compared to oxybutynin base, leading to a higher burst release. Accordingly, the entrapment of oxybutynin as a base was found to be more suitable. PLGA’s degradation generates a local acidification within the matrix, thereby increasing polymer chain mobility and pore generation, thus enhancing the drug release. As the pH increases, the fraction of protonated oxybutynin is reduced and the tendency of the drug to diffuse out is decreased, as was observed at a pH of 8 in the surrounding release medium. Due to the semi-solid structure of the implants during their formation until their degradation, it is analytically difficult to assess. A deeper understanding of potential drug/polymer interactions should be one focus in future studies.

A variety of intravesical drug delivery approaches have been investigated to prolong residence time and achieve sustained release. Thermoresponsive gels have been shown to be instilled with ease and to solidify at body temperature. However, these gels are susceptible to dilution and rapid elimination during micturition [33]. Microparticle suspensions have demonstrated potential for controlled release; nevertheless, particles may be expelled with urine, thereby limiting their effectiveness [2,33]. By providing localized, sustained release while minimizing systemic exposure and eliminating the need for surgical retrieval, ISFIs can directly confront the unique challenges associated with bladder drug administration and possess the potential to significantly enhance treatment outcomes and improve patient quality of life. ISFIs could offer distinct advantages over non-biodegradable implants, particularly regarding their ease of administration, customizable release profiles, and the elimination of surgical removal. Although this sustained release form offers a way of tailor-made site-specific and controlled drug delivery, further anatomical aspects need to be elucidated in more detail, such as high urine turnover and continuous bladder movement.

## 5. Conclusions

ISFIs, initially utilized predominantly for intramuscular delivery, have demonstrated potential for adaptation to intravesical administration, thereby addressing neurogenic detrusor overactivity. It is hypothesized that ISFI systems could represent a significant advancement in sustained intravesical drug delivery due to their biodegradability and the ability to form in situ. A smart choice of drug load and polymer grade enables the adjustment of both the initial burst and the long-term release. In addition, it can be concluded that the use of an alkaline drug increased the degradation of the polymer matrix, which could be further enhanced by the use of an acid-terminated PLGA, due to its increased hydrophilicity. Subsequent steps could also address an optimal treatment period for one single ISFI administration to further parallel implant degradation and full drug release. Additionally, patient safety concerns, such as the maximum drug load of the ISFIs, which can result in significant adverse effects when systemically available, must be considered.

## Figures and Tables

**Table 1:** Glass transition temperature of 502 and 502H neat and different implants after 7 d in artificial urine.

	502	502H
NEAT	39.0 °C ± 2.6 °C	42.5 °C ± 3.6 °C
30/0	23.9 °C ± 1.6 °C	18.3 °C ± 8.2 °C
30/1.5	17.9 °C ± 4.7 °C	16.3 °C ± 1.9 °C
30/3	14.9 °C ± 4.0 °C	13.1 °C ± 4.3 °C
30/7.5	6.5 °C ± 0.8 °C	7.5 °C ± 0.9 °C
30/15	<5 °C	<5 °C
15/0	23.2 °C ± 0.5 °C	16.8 °C ± 3.2 °C
15/15	<5 °C	<5 °C

## Data Availability

The original contributions presented in this study are included in the article/Supplementary Materials. Further inquiries can be directed to the corresponding author.

## Supplementary Materials

The following supporting information can be downloaded at: https://www.mdpi.com/article/10.3390/pharmaceutics17111369/s1.
