# Supplementary material for: In-Situ Forming Polyester Implants for Sustained Intravesical Oxybutynin Release
Source: Pharmaceutics. 2025 Oct 23;17(11):1369. doi: 10.3390/pharmaceutics17111369 (PMC12655623; doi:10.3390/pharmaceutics17111369)
Supplement: Supplementary file 1 [file pharmaceutics-17-01369-s001.zip › pharmaceutics-3899821-supplementary.pdf]

1. Supplementary

1.1. Comparison of the Salt with the Base Regarding the Drug Release

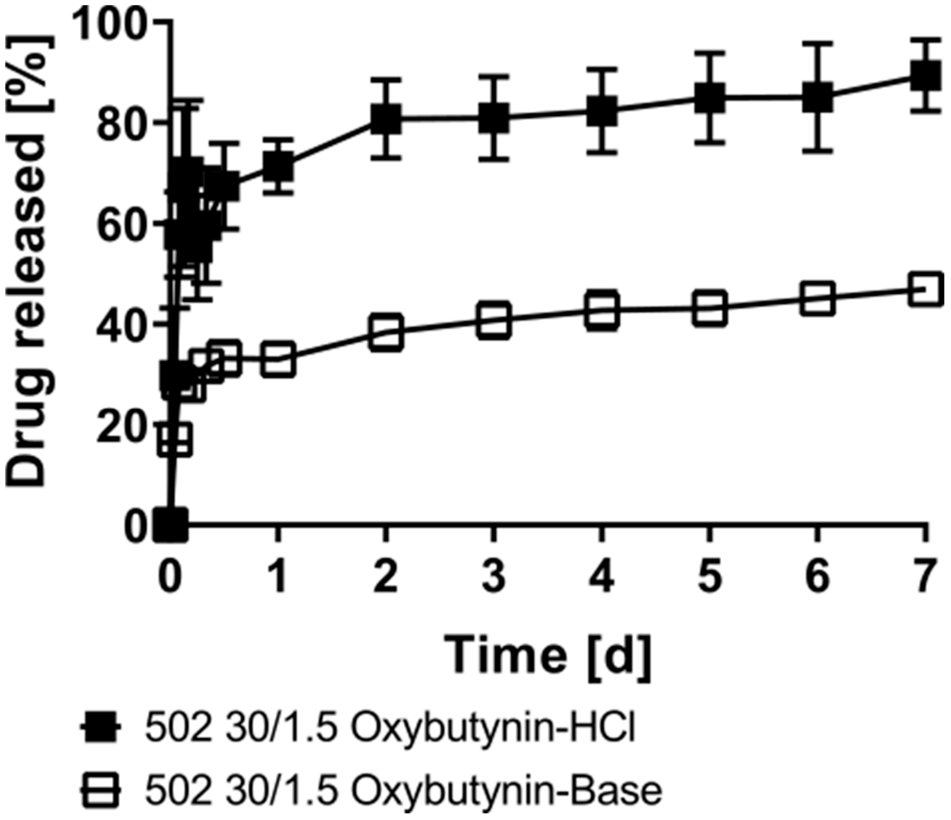

Figure S1: In-vitro drug release from of ISFI with a polymer load of 30 % and a drug load of 1.5 % was investigated in artificial urine pH 6 for 7 d. Oxybutynin-HCl was compared with Oxybutynin-Base (n = 3).

Oxybutynin hydrochloride was initially compared with its free base form to investigate the influence of it on the release from PLGA implants (Figure S1). The burst was twice as high for the hydrochloride, while controlled release phase was comparable.

1.2. Visualization of ISFI

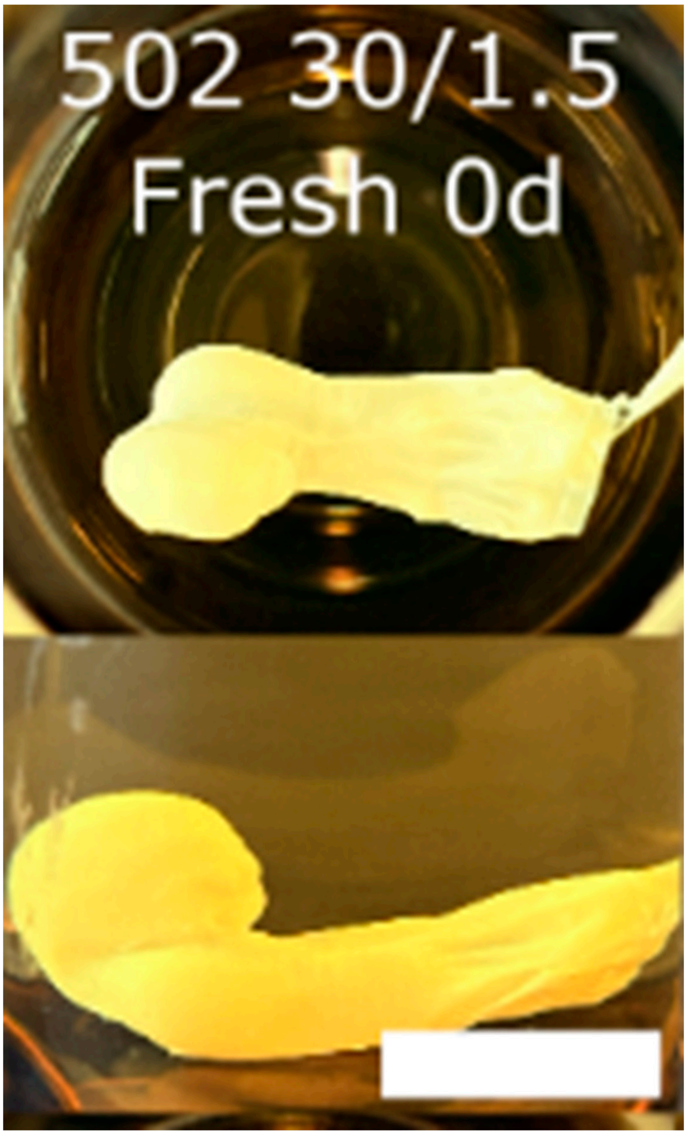

Figure S2: Visualization of ISFI 502 30/1.5 directly after injection in artificial urine pH 6. Scale bar represents 10mm.

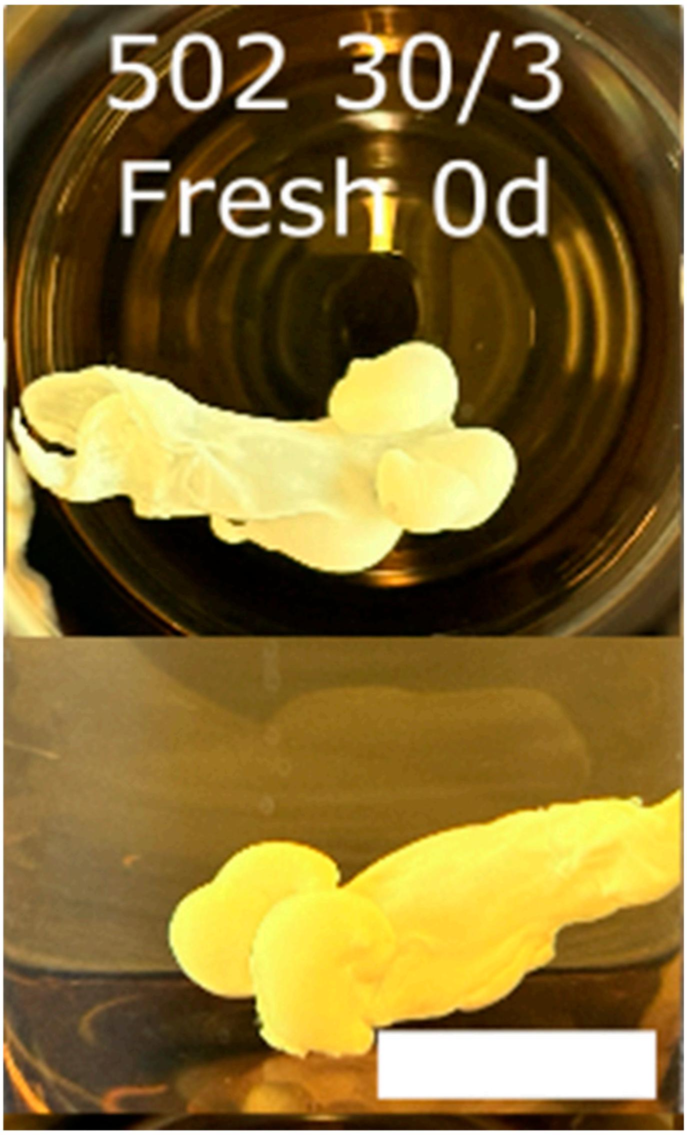

Figure S3: Visualization of ISFI 502 30/3 directly after injection in artificial urine pH 6. Scale bar represents 10mm.

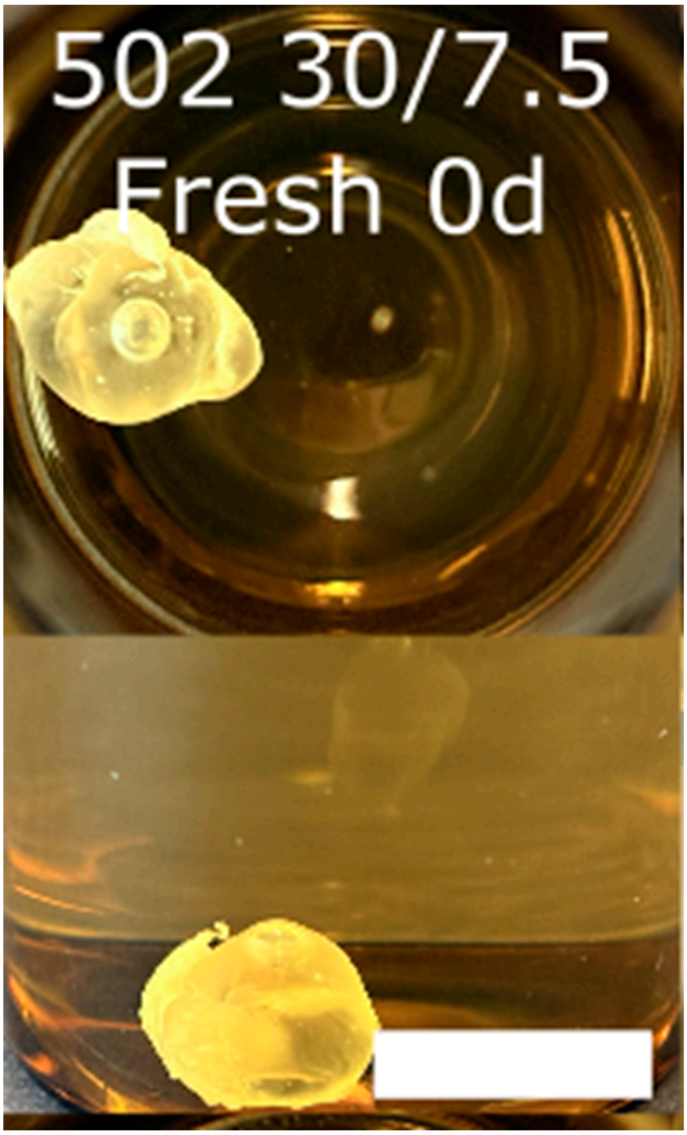

Figure S4: Visualization of ISFI 502 30/7.5 directly after injection in artificial urine pH 6. Scale bar represents 10mm.

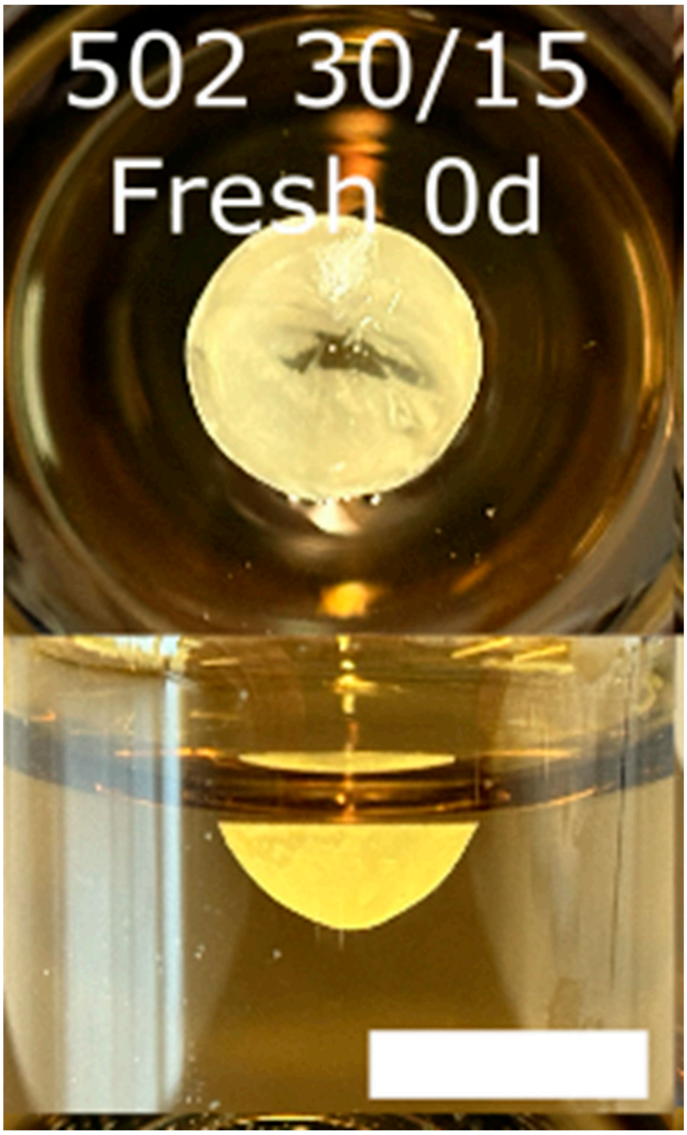

Figure S5: Visualization of ISFI 502 30/15 directly after injection in artificial urine pH 6. Scale bar represents 10mm.

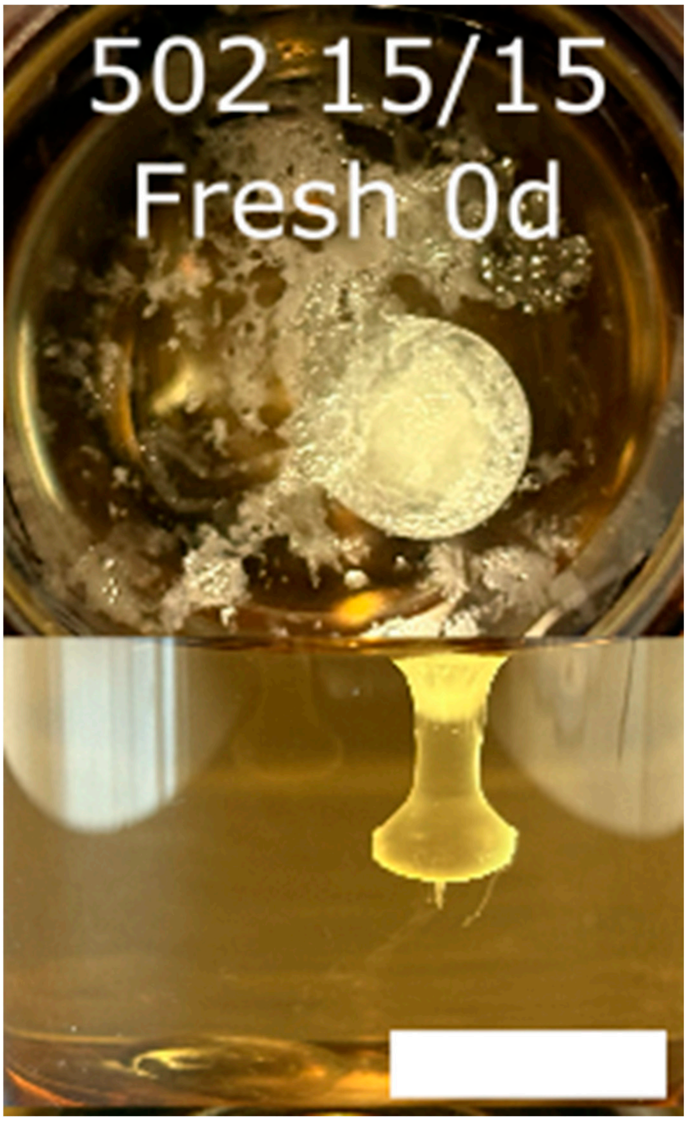

Figure S6: Visualization of ISFI 502 15/15 directly after injection in artificial urine pH 6. Scale bar represents 10mm.

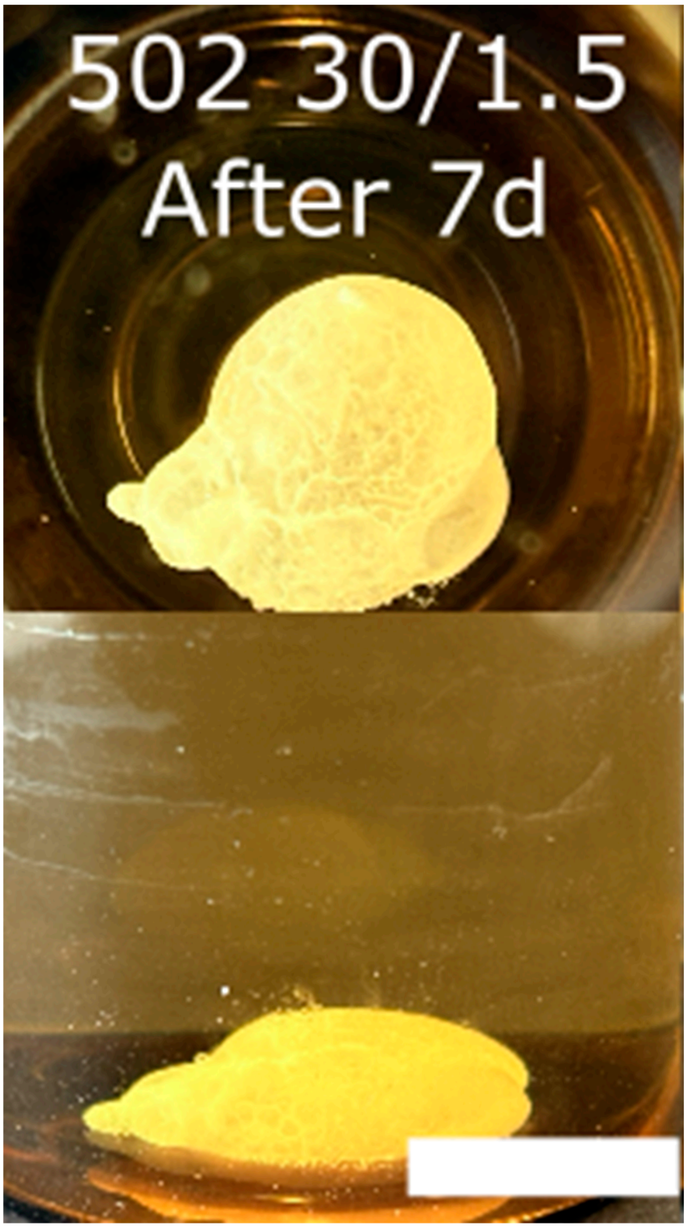

Figure S7: Visualization of ISFI 502 30/1.5 after 7d in artificial urine pH 6. Scale bar represents 10mm.

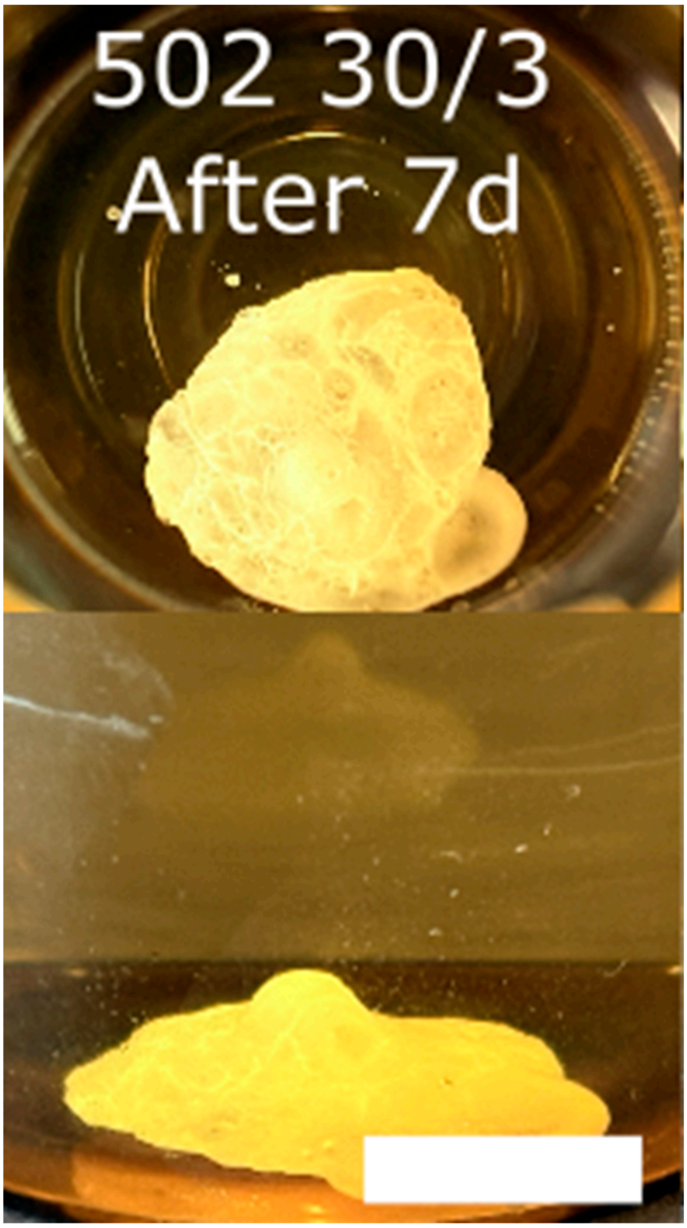

Figure S8: Visualization of ISFI 502 30/3 after 7d in artificial urine pH 6. Scale bar represents 10mm.

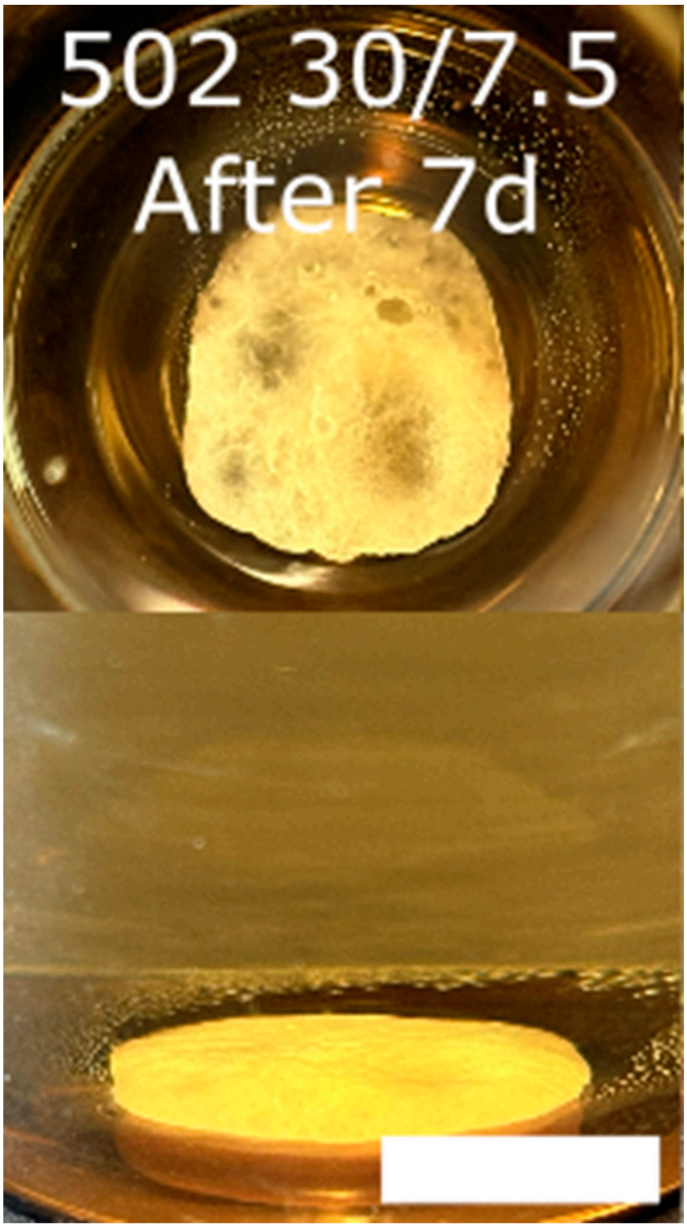

Figure S9: Visualization of ISFI 502 30/7.5 after 7d in artificial urine pH 6. Scale bar represents 10mm.

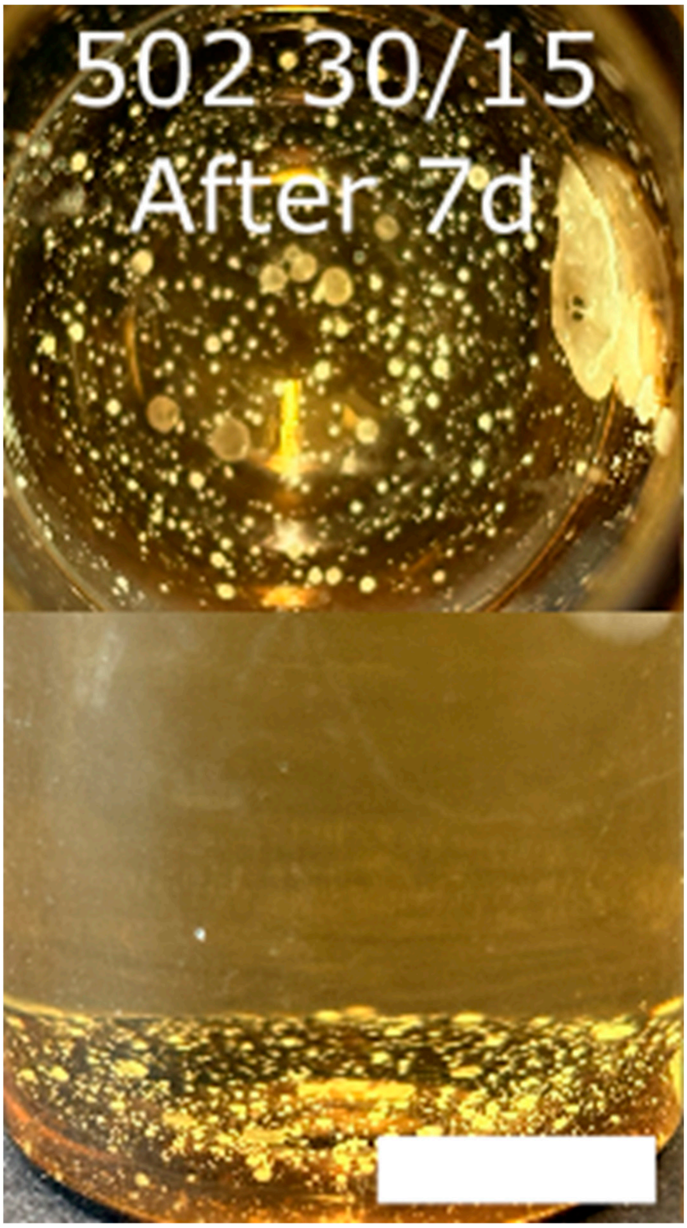

Figure S10: Visualization of ISFI 502 30/15 after 7d in artificial urine pH 6. Scale bar represents 10mm.

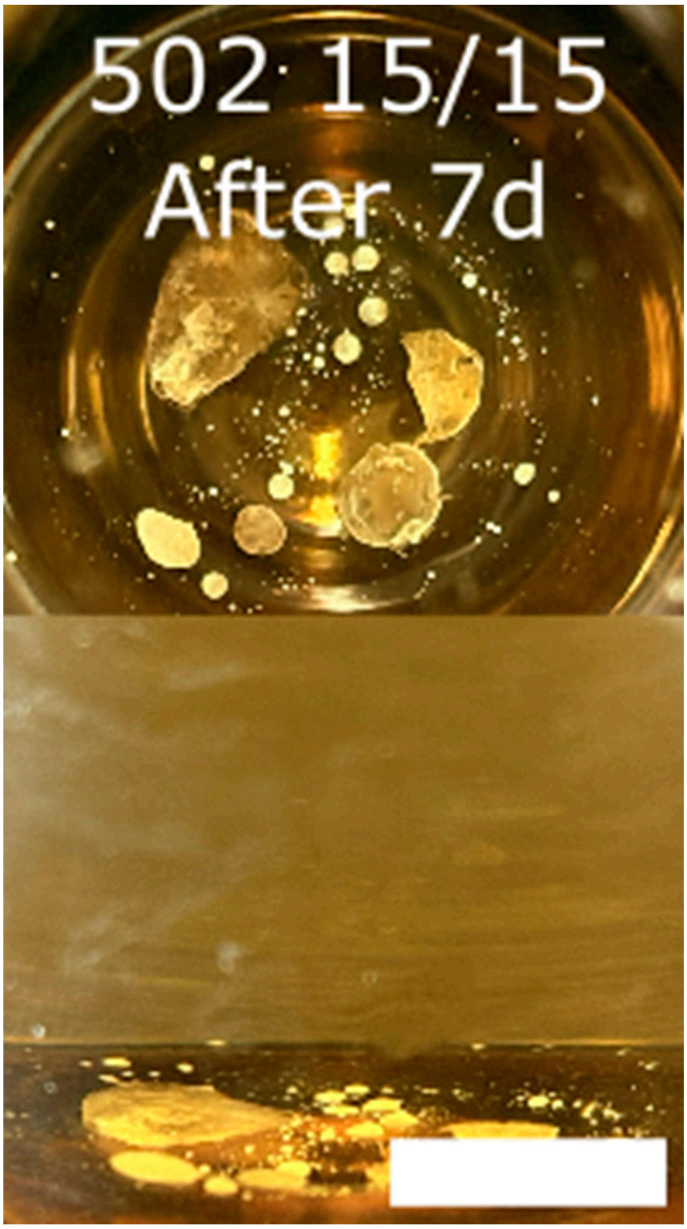

Figure S11: Visualization of ISFI 502 15/15 after 7d in artificial urine pH 6. Scale bar represents 10mm.

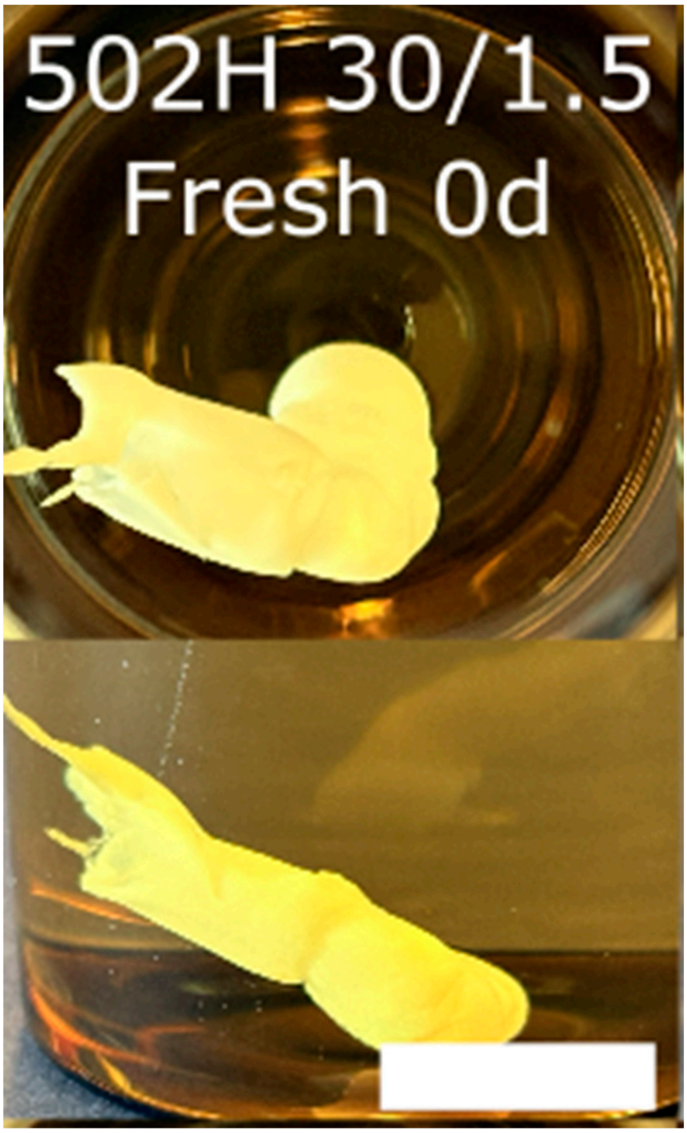

Figure S12: Visualization of ISFI 502H 30/1.5 directly after injection in artificial urine pH 6. Scale bar represents 10mm.

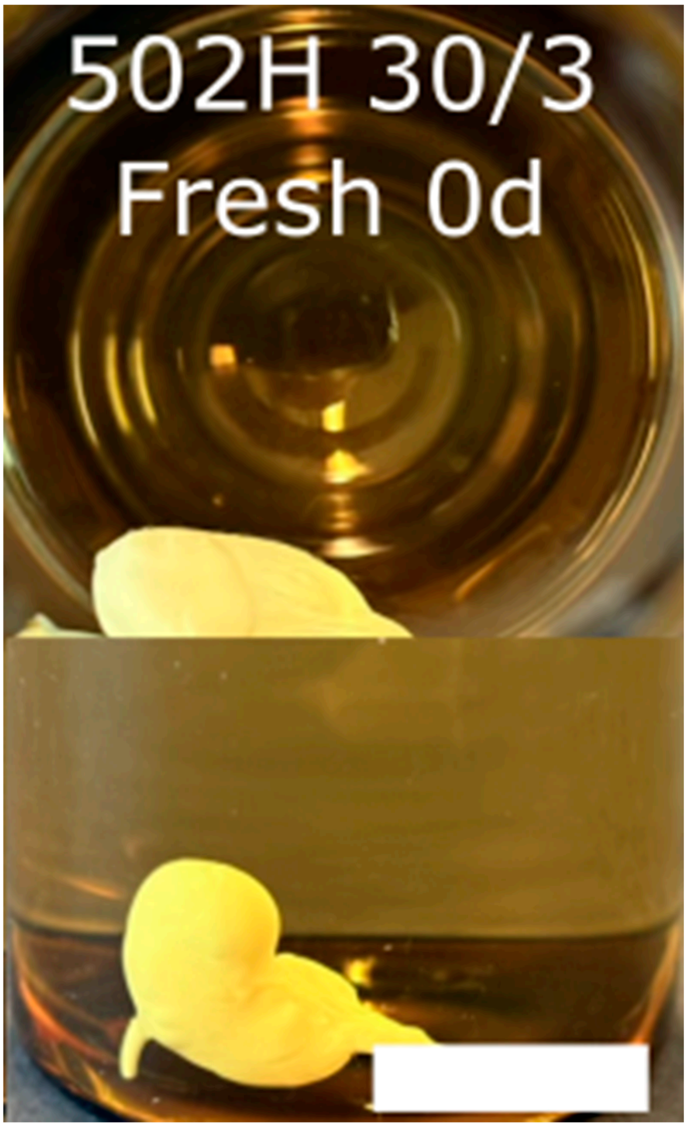

Figure S13: Visualization of ISFI 502H 30/3 directly after injection in artificial urine pH 6. Scale bar represents 10mm.

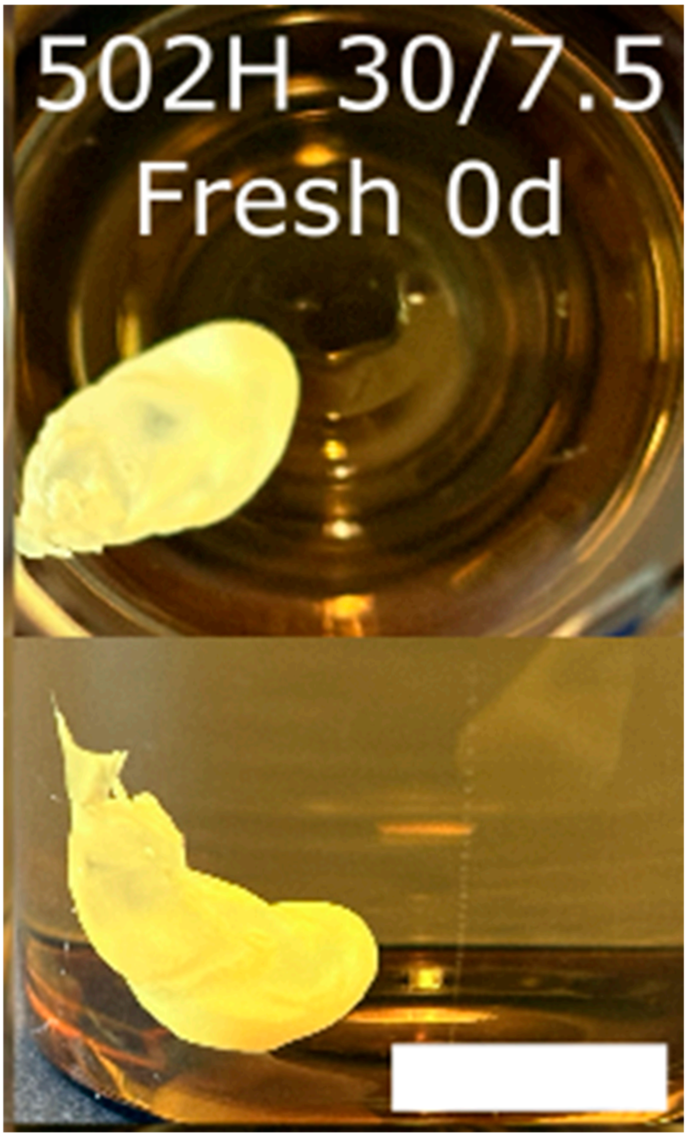

Figure S14: Visualization of ISFI 502H 30/7.5 directly after injection in artificial urine pH 6. Scale bar represents 10mm.

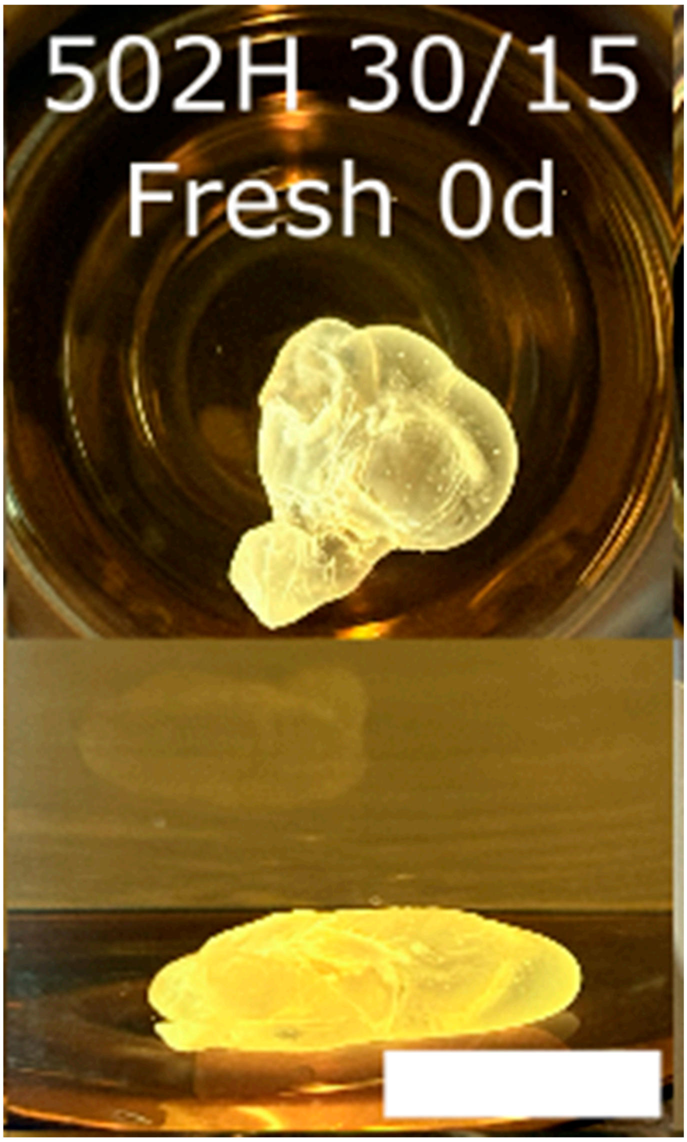

Figure S15: Visualization of ISFI 502H 30/15 directly after injection in artificial urine pH 6. Scale bar represents 10mm.

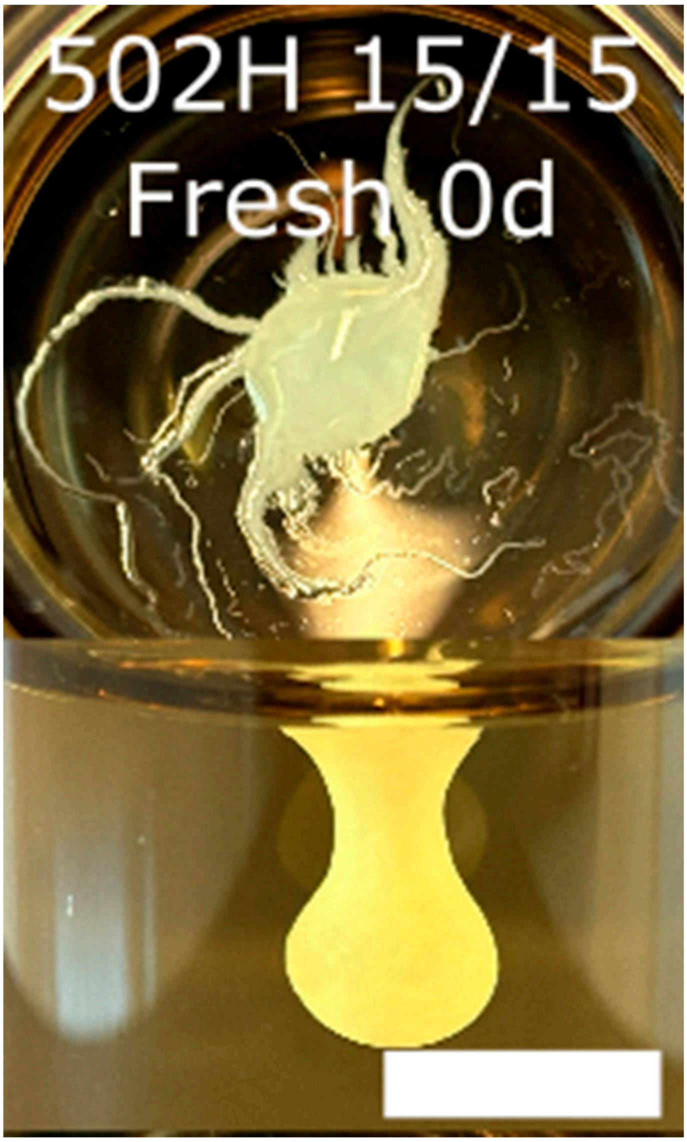

Figure S16: Visualization of ISFI 502H 15/15 directly after injection in artificial urine pH 6. Scale bar represents 10mm.

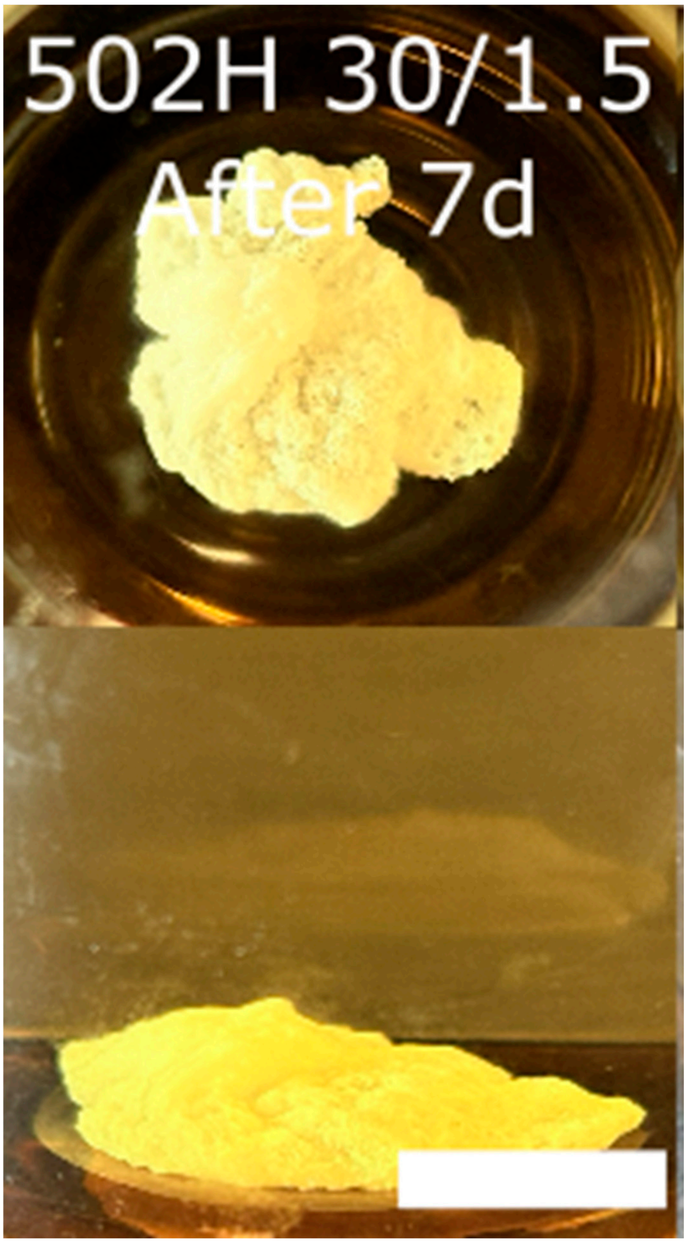

Figure S17: Visualization of ISFI 502H 30/1.5 after 7d in artificial urine pH 6. Scale bar represents 10mm.

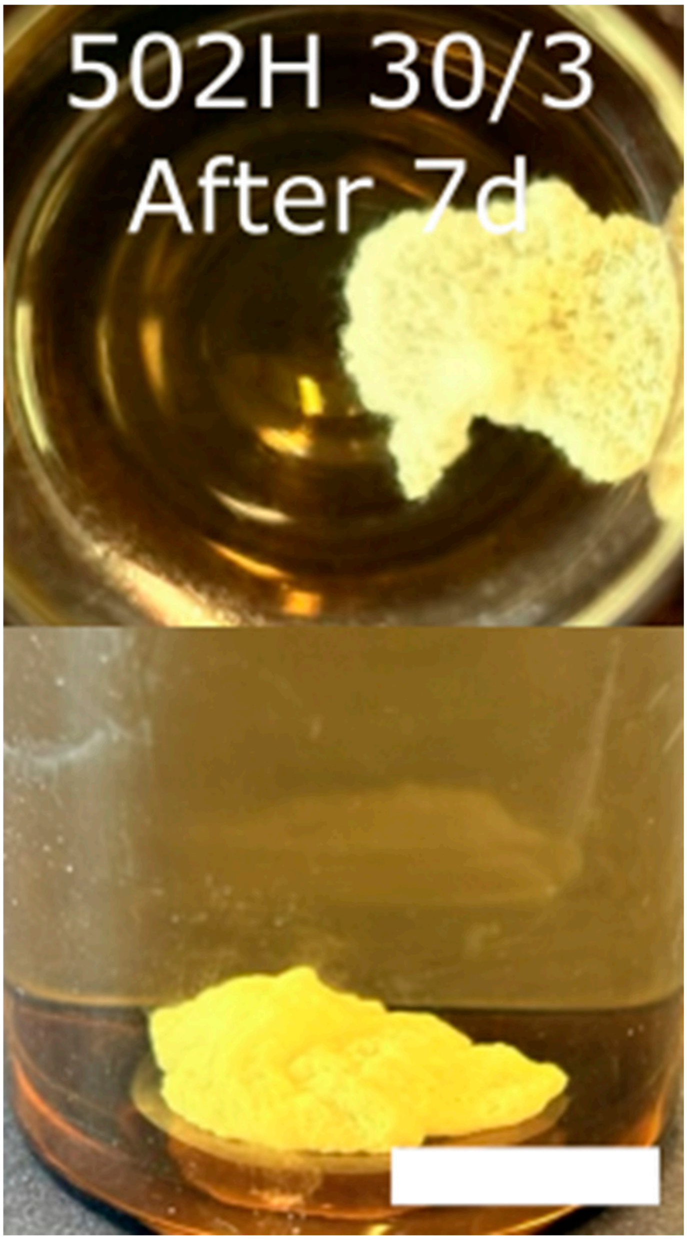

Figure S18: Visualization of ISFI 502H 30/3 after 7d in artificial urine pH 6. Scale bar represents 10mm.

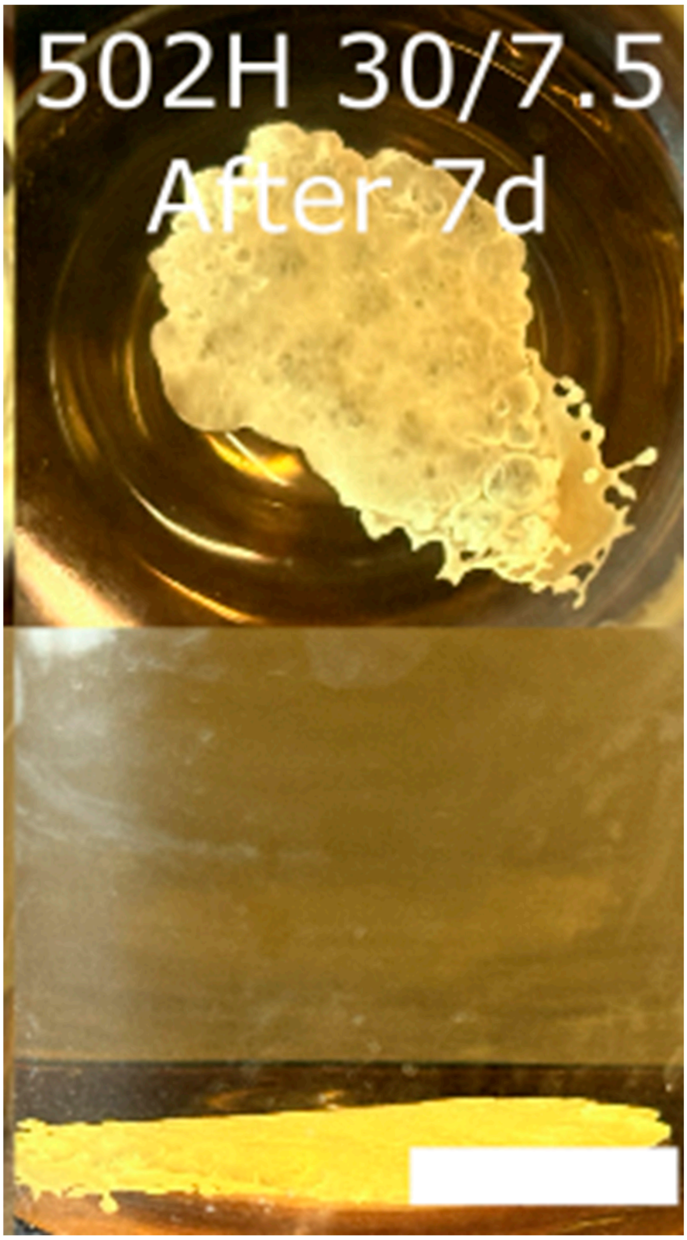

Figure S19: Visualization of ISFI 502H 30/7.5 after 7d in artificial urine pH 6. Scale bar represents 10mm.

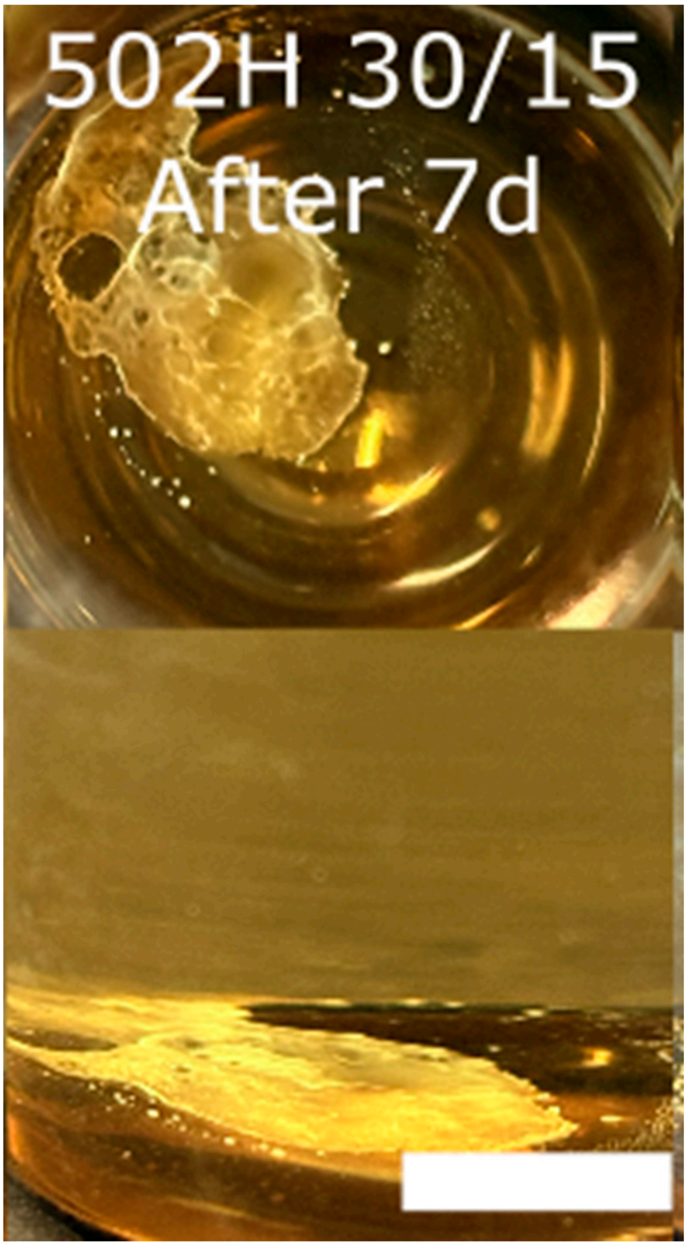

Figure S20: Visualization of ISFI 502H 30/15 after 7d in artificial urine pH 6. Scale bar represents 10mm.

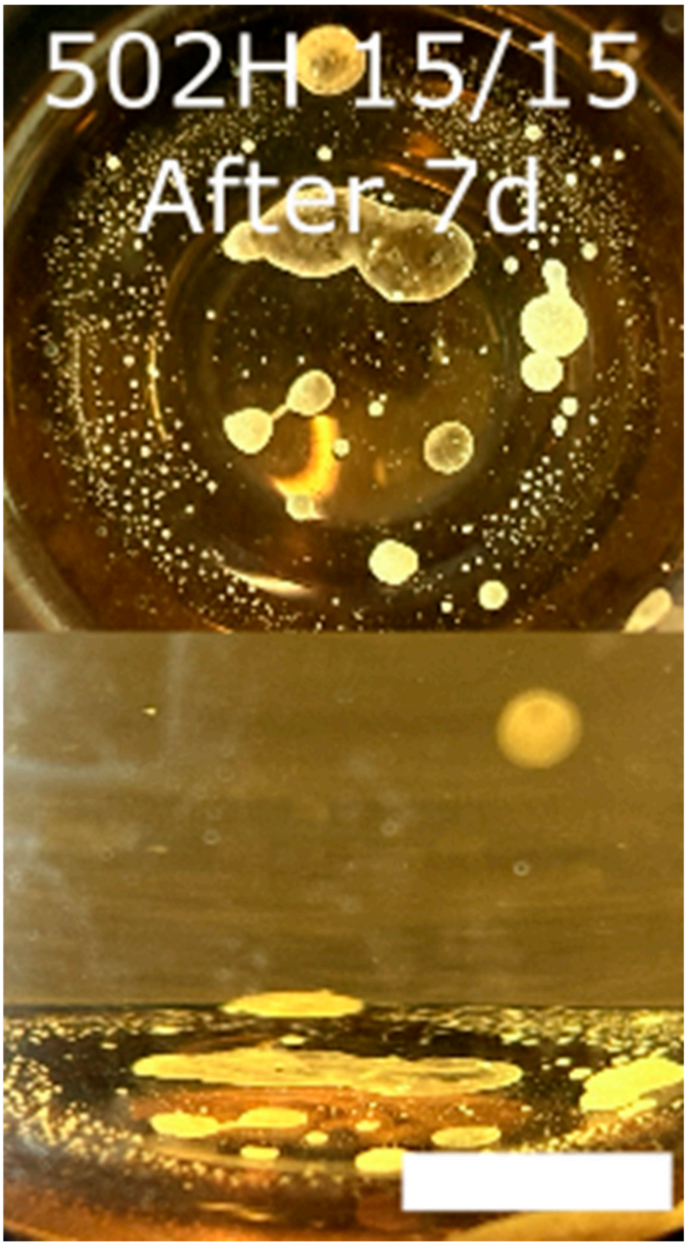

Figure S21: Visualization of ISFI 502H 15/15 after 7d in artificial urine pH 6. Scale bar represents 10mm.
